# Chronic Oral Anticoagulation Therapy and Prognosis of Patients Admitted to Hospital for COVID-19: Insights from the HOPE COVID-19 Registry

**DOI:** 10.1155/2022/7325060

**Published:** 2022-05-26

**Authors:** José Miguel Rivera-Caravaca, Iván J. Núñez-Gil, Gregory Y. H. Lip, Aitor Uribarri, María C. Viana-Llamas, Adelina Gonzalez, Alex F. Castro-Mejía, Berta Alonso González, Emilio Alfonso, Juan Fortunato García Prieto, Chiara Cavallino, Bernardo Cortese, Gisela Feltes, Inmaculada Fernández-Rozas, Jaime Signes-Costa, Jia Huang, Marcos García Aguado, Martino Pepe, Rodolfo Romero, Enrico Cerrato, Víctor Manuel Becerra-Muñoz, Sergio Raposeiras Roubin, Francesco Santoro, Rodrigo Bagur, Luciano Sposato, Ibrahim El-Battrawy, Alvaro López Masjuan, Antonio Fernandez-Ortiz, Vicente Estrada, Carlos Macaya, Francisco Marín

**Affiliations:** ^1^Department of Cardiology, Hospital Clínico UniversitarioVirgen de la Arrixaca, University of Murcia, Instituto Murciano de Investigación Biosanitaria (IMIB-Arrixaca), CIBERCV, Murcia, Spain; ^2^Liverpool Centre for Cardiovascular Science, University of Liverpool, Liverpool Heart & Chest Hospital, Liverpool, UK; ^3^Hospital Clínico San Carlos Universidad Complutense de Madrid, Instituto de Investigación Sanitaria del Hospital Clínico San Carlos (IdISSC), Madrid, Spain; ^4^Department of Clinical Medicine, Aalborg University, Aalborg, Denmark; ^5^Hospital Clínico Universitario de Valladolid, Valladolid, Spain; ^6^Hospital Universitario Guadalajara, Guadalajara, Spain; ^7^Hospital Universitario Infanta Sofía, San Sebastian de los Reyes, Madrid, Spain; ^8^Hospital General del Norte de Guayaquil IESS Los Ceibos, Guayaquil, Ecuador; ^9^Hospital Universitario la Paz, Madrid, Spain; ^10^Instituto de Cardiología y Cirugía Cardiovascular, La Habana, Cuba; ^11^Hospital de Manises, Valencia, Spain; ^12^Sant' Andrea Hospital, Vercelli, Italy; ^13^San Carlo Clinic, Milano, Italy; ^14^Hospital Nuestra Señora de América, Madrid, Spain; ^15^Hospital Severo Ochoa, Leganés, Spain; ^16^Hospital Clínico Universitario, INCLIVA, Universidad de Valencia, Valencia, Spain; ^17^The Second People's Hospital of Shenzhen, Shenzhen, China; ^18^Hospital Universitario Puerta de Hierro, Majadahonda, Spain; ^19^Azienda Ospedaliero Universitaria Consorziale Policlinico di Bari, Bari, Italy; ^20^Hospital Universitario de Getafe, Madrid, Spain; ^21^San Luigi Gonzaga University Hospital, Rivoli, Turin, Italy; ^22^Unidad de Gestión Clínica Área del Corazón, Instituto de Investigación Biomédica de Málaga (IBIMA), Hospital Universitario Virgen de la Victoria, Universidad de Málaga (UMA), Centro de Investigación Biomédica en Red Enfermedades Cardiovasculares (CIBERCV), Málaga, Spain; ^23^University Hospital Álvaro Cunqueiro, Vigo, Spain; ^24^Azienda Sanitaria Locale della Provincia di Barletta-Andria-Trani, Andria, Italy; ^25^Department of Medical and Surgery Sciences, University of Foggia, Foggia, Italy; ^26^London Health Sciences Centre, London, Ontario, Canada; ^27^Schulich School of Medicine and Dentistry, Western University, London, Ontario, Canada; ^28^Lawson Health Research Institute, London, Ontario, Canada; ^29^Robarts Research Institute, London, Ontario, Canada; ^30^University of Mannheim, Mannheim, Germany; ^31^Hospital Universitario Juan Ramón Jiménez, Huelva, Spain

## Abstract

**Background:**

Most evidence regarding anticoagulation and COVID-19 refers to the hospitalization setting, but the role of oral anticoagulation (OAC) before hospital admission has not been well explored. We compared clinical outcomes and short-term prognosis between patients with and without prior OAC therapy who were hospitalized for COVID-19.

**Methods:**

Analysis of the whole cohort of the HOPE COVID-19 Registry which included patients discharged (deceased or alive) after hospital admission for COVID-19 in 9 countries. All-cause mortality was the primary endpoint. Study outcomes were compared after adjusting variables using propensity score matching (PSM) analyses.

**Results:**

7698 patients were suitable for the present analysis (675 (8.8%) on OAC at admission: 427 (5.6%) on VKAs and 248 (3.2%) on DOACs). After PSM, 1276 patients were analyzed (638 with OAC; 638 without OAC), without significant differences regarding the risk of thromboembolic events (OR 1.11, 95% CI 0.59–2.08). The risk of clinically relevant bleeding (OR 3.04, 95% CI 1.92–4.83), as well as the risk of mortality (HR 1.22, 95% CI 1.01–1.47; log-rank *p* value = 0.041), was significantly increased in previous OAC users. Amongst patients on prior OAC only, there were no differences in the risk of clinically relevant bleeding, thromboembolic events, or mortality when comparing previous VKA or DOAC users, after PSM.

**Conclusion:**

Hospitalized COVID-19 patients on prior OAC therapy had a higher risk of mortality and worse clinical outcomes compared to patients without prior OAC therapy, even after adjusting for comorbidities using a PSM. There were no differences in clinical outcomes in patients previously taking VKAs or DOACs. This trial is registered with NCT04334291/EUPAS34399.

## 1. Introduction

Vascular inflammation, hypercoagulable state, and endothelial dysfunction have been described in patients with severe acute respiratory syndrome coronavirus-2 (SARS-CoV-2) infection [1, 2]. As a result, thromboembolic complications are common in patients with coronavirus disease 2019 (COVID-19) [3–5]. Therefore, antithrombotic therapy, particularly anticoagulation, gained attention in the context of COVID-19. Indeed, some studies demonstrated that anticoagulation may be associated with improved outcomes among patients with COVID-19 [6, 7]. However, most of the evidence in relation to anticoagulation and COVID-19 refer to the acute hospitalization setting, whereas the role of stable oral anticoagulation (OAC) therapy before the admission for COVID-19 has not been well explored.

One study suggested a protective role of chronic direct-acting OAC (DOAC) therapy in elderly patients with COVID-19 [8]. In a preliminary analysis of the International COVID-19 Clinical Evaluation (HOPE COVID-19) Registry published previously, we observed that COVID-19 patients on OAC therapy at hospital admission had lower survival and higher mortality risk compared to patients without prior OAC [9].

In the present study, we aimed to compare clinical outcomes and in-hospital prognosis between patients on prior OAC therapy and patients not on OAC therapy who were admitted for COVID-19 and enrolled in the HOPE COVID-19 Registry, using a propensity score matching (PSM) approach. Second, we aimed to compare clinical outcomes and prognosis between patients on vitamin K antagonist (VKA) therapy and patients on DOACs before admission.

## 2. Methods

A detailed description of the HOPE COVID-19 Registry has been published elsewhere [10, 11]. Briefly, the HOPE COVID-19 is an ambispective international registry, real-life cohort “all comers” type, including more than 8100 patients from 9 countries (Canada, China, Chile, Colombia, Cuba, Ecuador, Germany, Italy, and Spain). The study was an initiative without conflicts of interest, no financial remuneration, and methodological support from the Institute for the Improvement of Health Care (IMAS) foundation (Madrid, Spain).

All patients discharged (deceased or alive) after hospital admissions for COVID-19 were suitable for the study. There were no exclusion criteria, except for patients' explicit refusal to participate. The first patient was included in February 2020. Clinical and demographic data were collected at inclusion and during the hospitalization in an anonymized database presented in the electronic format, to be filled in at each participating center (NCT04334291/EUPAS34399).

Reporting of the study conforms to broad EQUATOR guidelines. The study was performed according to the ethical principles of Declaration of Helsinki and Good Clinical Practice Guidelines and has been approved by Ethics Research Committee from the Hospital Clínico San Carlos (Madrid, Spain) (20/241-E) and the Spanish Agency for Medicines and Health Products (EPA-0D). Given the anonymous characteristics of the registry and the health alarm situation generated by the virus, in principle, written informed consent was waived. However, at least verbal authorization from the patient (or familiar or caregiver, when unavailable) was required.

### 2.1. Laboratory Analyses

Laboratory parameters were considered elevated as defined by local laboratory cutoff levels. However, the HOPE COVID-19 Registry protocol suggested the following as “elevated:” for D-dimer (≥0.5 mg/L), for procalcitonin (≥0.5 ng/mL), for C-reactive protein (≥10 mg/L), for troponins (>99th percentile), for transaminases (≥40 U/L), for ferritin (≥336 ng/mL), and for lactate dehydrogenase (≥280 U/L).

### 2.2. Study Outcomes

The primary endpoint for this analysis was in-hospital all-cause mortality. Any thrombotic/thromboembolic event and any clinically relevant bleeding were the secondary outcomes. Bleeding was defined as “relevant” at the discretion of the attending medical team and classified using the BARC bleeding score as type 2, 3, or 5.

Although not classified as primary or secondary outcomes, other adverse events during hospitalization were recorded, including renal failure, respiratory insufficiency, upper respiratory tract infection, heart failure, sepsis, and systemic inflammatory response syndrome (SIRS).

Local researchers identified, confirmed, and recorded all adverse events. The clinical management was decided, in all cases, by the attending team and researchers had no role in this point.

### 2.3. Statistical Analysis

Quantitative variables were expressed as mean ± standard deviation (SD) or median and interquartile range (IQR), as appropriate according to the Kolmogorov–Smirnov test, whilst categorical variables were expressed as absolute frequencies and percentages. Pearson's chi-squared test was used to compare proportions. Differences between two groups regarding a quantitative variable were tested with Student's *t* or the Mann–Whitney *U* tests, as appropriate if normally or not normally distributed.

To compare the risk of the study outcomes among patients on prior OAC therapy and patients without prior OAC therapy, we conducted a propensity score matching (PSM) adjusting for demographics and baseline comorbidities. The risk of the study outcomes among patients on prior VKA therapy or DOACs was also evaluated by another PSM. In both PSMs, those variables that were significantly different between both cohorts were included in the model to adjust for differences. Patients were matched 1 : 1 across each cohort on a propensity score generated by logistic regressions using the nearest neighbour technique without replacement with a maximum caliper of 0.2, thus avoiding at least 98% of the bias due to the measured confounders. The value of absolute standardized mean difference <10% indicated balance of matched cohorts [12, 13].

Survival analyses by Kaplan–Meier estimates were performed after PSM to assess differences in event-free survival of the primary outcome depending on the use (or not) of prior OAC therapy and depending on the use of prior VKA or DOAC therapy. The risk of suffering from the primary outcome was assessed by Cox proportional hazard regression, and results were reported as hazard ratio (HR) with 95% confidence interval (CI). The risk of suffering from other study outcomes was investigated by logistic regression analyses, since the exact date for these events was not recorded. In these analyses, results were reported as odds ratio (OR) with 95% confidence interval (CI).

Two-sided *p* values <0.05 were accepted as statistically significant. Statistical analyses were performed using SPSS v. 24.0 (SPSS, Inc., Chicago, IL, USA) and MedCalc v. 16.4.3 (MedCalc Software bvba, Ostend, Belgium) for Windows.

## 3. Results

A cohort of 8168 patients was included. After excluding patients with insufficient or not reliable data on previous OAC, 7698 patients remained in the study (4500 (58.5%) male; median age of 65 (IQR 51–77) years). Of these, 675 (8.8%) were on OAC therapy at hospital admission, 427 (5.6%) were on VKAs, and 248 (3.2%) were on DOACs.

### 3.1. Outcomes on Prior OAC Therapy

In the overall cohort of 7698 patients, we found that patients on prior OAC therapy were less commonly admitted in the intensive care unit (ICU) compared to patients not previously taking OACs (6.7% vs. 10.1%, *p*=0.004). During hospitalization, the prognosis of patients on prior OAC therapy was also poor, and these patients had more incident heart failure, renal failure, sepsis, and SIRS (all with *p* value <0.001). As expected, the risk of any clinically relevant bleeding in patients with previous OAC therapy was higher compared to patients not taking OAC previously (11.6% vs. 3.4%, *p* < 0.001; OR 3.71, 95% CI 2.83–4.85), without differences in terms of thromboembolic events (3.1% vs. 2.7%, *p*=0.493). The risk of mortality was found to be significantly increased in patients on prior OAC therapy (39.1% vs. 17.0%, *p* < 0.001; HR 2.45, 95% CI 2.14–2.79); however, there were significant differences between patients on prior and not on prior OAC in terms of several comorbidities. We therefore performed PSM to adjust these analyses (Table 1).

After PSM, 1276 patients remained in the study (638 : 638 paired comparisons), with no significant differences regarding admission to the ICU in patients on prior OAC compared to patients not previously taking OACs (6.9% vs. 6.3%, *p*=0.652). The prognosis of patients on prior OAC therapy during hospitalization was still poor even after adjustment, and these patients suffered more commonly from heart failure, renal failure, and SIRS (all with *p* value <0.05). No significant differences were found in terms of respiratory insufficiency (67.2% vs. 64.7%; *p*=0.280), upper respiratory tract infection (13.9% vs. 14.1%; *p*=0.987), or sepsis (15.0% vs. 12.1%; *p*=0.299) (Table 2).

**Figure 1:**
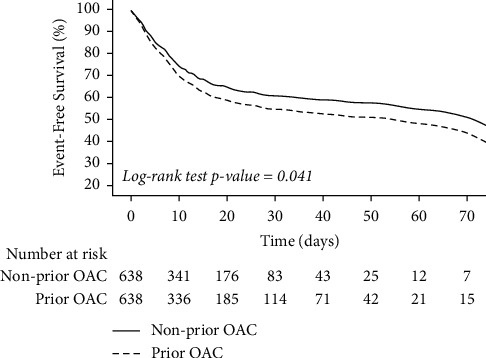
Comparison of survival curves between patients on prior OAC and nonprior OAC. Solid line, nonprior OAC; dashed line, prior OAC.

Similar to the finding observed before PSM, the risk of any clinically relevant bleeding was higher in patients with previous OAC therapy compared to patients not taking OAC previously (11.4% vs. 4.1%, *p* < 0.001; OR 3.04, 95% CI 1.92–4.83), without differences in the risk of thromboembolic events (3.3% vs. 3.0%, *p*=0.748; OR 1.11, 95% CI 0.59–2.08). There was increased mortality in patients who were on previous OAC therapy in comparison to patients who were not on previous OAC (38.1% vs. 30.9%, *p*=0.007), with a significantly higher risk of death (HR 1.22, 95% CI 1.01–1.47), also confirmed by the Kaplan–Meier analysis (log-rank *p* value = 0.041) (Figure 1). There were no differences between patients on prior or non-prior OAC therapy regarding specific causes of death (cardiovascular death: 2.6% vs. 2.5%; respiratory-related: 59.7% vs. 62.9%; SIRS-related: 4.9% vs. 3.6%; sepsis-related: 3.3% vs. 7.6%; other reasons or combined causes of death: 29.6% vs. 23.4%; *p*=0.187).

### 3.2. Impact of OAC Type

In patients on prior OAC therapy, we observed significant differences regarding age and comorbid conditions between patients who were previously taking VKAs and those who were on prior DOAC therapy. We performed another PSM to balance these characteristics. This analysis demonstrated no differences in the remaining 464 subjects: 232 on VKAs and 232 on DOACs, as given in Table 3.

With this matched cohort, the rate of ICU admission between patients on VKAs (14, 6.0%) and patients on DOACs (14, 6.0%) was similar (*p*=1.000). There were no differences in terms of respiratory insufficiency, heart failure, development of renal failure, upper respiratory tract infection, sepsis, or SIRS, in patients on prior VKAs or DOACs (all *p* > 0.005) (Supplementary Table 1).

**Figure 2:**
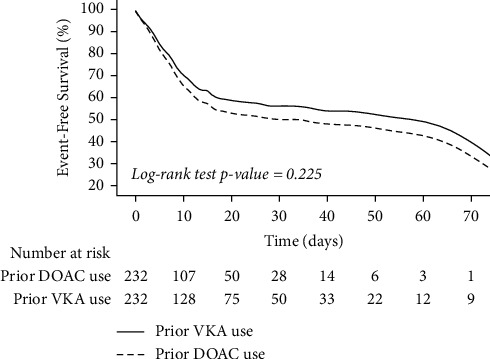
Comparison of survival curves between patients on prior VKAs or DOACs. Solid line, prior VKA use; dashed line, prior DOAC use.

No significant differences in the incidences of clinically relevant bleeding or thromboembolic events were observed in patients previously taking VKAs compared to DOACs (1.01 vs. 0.83 per 100 patient-days (*p*=0.458) and 0.29 vs. 0.11 per 100 patient-days (*p*=0.127), respectively) (Supplementary Table 1). Mortality rate between previous VKA and DOAC users was also similar (37.9% vs. 39.7%, *p*=0.703), with a non-significant difference in mortality risk amongst previous VKA users (HR 0.84, 95% CI 0.62–1.12; *p*=0.233) (Figure 2).

### 3.3. Anticoagulation Management during Hospitalization

Regarding anticoagulation during hospitalization in the PSM cohort of previous vs. no previous OAC, most patients not taking OAC previously were prescribed heparin (79.9%, 382/478) and 19.2% (92/478) did not receive anticoagulation. In patients who were previously on OAC, 65.5% (330/504) were switched to heparin, 25% (126/504) continued on OAC, and 9.5% (48/504) did not receive any anticoagulation therapy. These proportions were significantly different (*p* < 0.001).

In the PSM cohort of previous VKA vs. DOAC, most patients under either therapy received heparin during hospitalization, without differences between drug families (116 vs. 114; *p*=0.567). Those patients who were maintained on OAC during admission were predominately treated with the same OAC that they were before (95.5% for previous VKAs users and 93.2% for previous DOACs users; *p* < 0.001).

## 4. Discussion

In this study of the HOPE COVID-19 Registry, including a large cohort of patients hospitalized for COVID-19, we demonstrate that the risk of in-hospital worse clinical outcomes was higher in patients with prior OAC therapy, even after adjustment by a PSM. Importantly, this study population showed a 22% higher risk of mortality and bleeding, without significant differences in the prognosis with regard to the particular anticoagulant drug, i.e., VKAs versus DOACs.

Anticoagulation in the context of COVID-19 has been widely debated, with some studies showing that prophylactic and therapeutic anticoagulation might reduce mortality in hospitalized COVID-19 patients [14]. Patients who received high-intensity prophylactic anticoagulation have a downtrend in D-dimer levels and improved 30-day mortality [15]. Indeed, a cross-sectional analysis showed that anticoagulation use was associated with delayed death, both at prophylactic (HR 0.29, 95% CI 0.15–0.58; *p* < 0.001) and therapeutic doses (HR 0.15, 95% CI 0.07–0.32; *p* < 0.001), compared with no anticoagulation [16]. In contrast, one retrospective analysis of hospitalized COVID-19 patients suggested that therapeutic anticoagulation provided no mortality benefit over thromboprophylaxis, independently of comorbidities or disease severity, and more adverse events were observed with therapeutic anticoagulation [17]. On the other hand, a large cohort study simulating an intention-to-treat clinical trial analyzed the effect on mortality of anticoagulation therapy chosen in the first 48 hours of hospitalization showing that patients with moderate or severe illness benefited from anticoagulation and that apixaban had a similar efficacy to enoxaparin in decreasing mortality amongst these patients [18]. Another study showed that hospitalized COVID-19 patients suffered from more bleeding events in those on low-molecular-weight heparin (LMWH) compared to DOACs, and DOAC use may be associated with better survival and lower invasive respiratory support rate compared to LMWH [19]. Given such contradictory observations, there are a number of studies and clinical trials with the aim to assess the role of antithrombotic therapy on mortality and thromboembolic events [20–26].

OAC management in the setting of the COVID-19 pandemic is even more complex. VKAs have the limitation of routine monitoring and dose adjusting for maintaining good quality of anticoagulation. One study demonstrated a significant increase in high INR results during the COVID-19 pandemic, the majority of them after the introduction of a lockdown [27]. In addition, patients on VKA hospitalized with SARS-CoV-2 showed greater instability of PT INR due to the inflammatory state and the interactions with numerous drugs. On the other hand, DOACs avoid some of the VKA limitations, but DOAC-treated patients have an increase in DOAC plasma levels when treated with antiviral drugs for COVID-19 [28]. For these reasons, some groups have suggested replacing OAC with parenteral heparin during hospitalization to avoid the risk of over/under treatment [29, 30]. Nevertheless, other authors suggested that the indications for antiplatelet/anticoagulant use (prevention, prophylaxis, and therapy) should be guided by the clinical context and the COVID-19 severity and not based on a systematic change per protocol in all patients [31, 32].

Nevertheless, most of the evidence focused on hospitalized patients, whereas the potential effect of chronic antithrombotic therapies in COVID-19 progression and prognosis remains uncertain. The pathophysiology underlying the prothrombotic state elicited by SARS-CoV-2 outlines possible protective mechanisms of antithrombotic therapy for this viral disease. In particular, aspirin and FXa inhibitors have been postulated as potential prophylactic and therapeutic treatment for high-risk patients with COVID-19 [31, 33]. Unsurprisingly, ongoing clinical trials are comparing the effectiveness and safety of apixaban, aspirin, and rivaroxaban versus heparin, placebo, and other therapies on progression, arterial, and venous thromboembolic events and mortality in patients with COVID-19 not yet admitted to hospital [34–36].

To date, data in this particular context are scarce and limited, with positive, negative, and neutral results. One small study in an Italian cohort of elderly patients with COVID-19 concluded that chronic DOAC intake was an independent parameter associated with a decreased mortality risk (HR 0.38, 95% CI 0.17–0.58; *p*=0.010) [8]. Similarly, another study in Italy showed that elderly patients with COVID-19 on chronic OAC treatment for atrial fibrillation had lower all-cause mortality rate ratio compared to their PSM non-anticoagulated counterpart [37]. However, Sivaloganathan et al. demonstrated that patients taking antithrombotic therapy (anticoagulant or antiplatelet agents) at the time of infection with COVID-19 did not have a significantly different mortality risk to those patients not taking these drugs [38]. Another study showed no difference in the risk of acute respiratory distress syndrome at admission or death during hospitalization between COVID-19 patients treated or not with antiplatelets or anticoagulants preadmission [39]. Likewise, anticoagulant use pre-COVID-19 diagnosis was not associated with a decreased risk for all-cause mortality, mechanical ventilation, or hospital admission in a study from the New York City health system, suggesting that previous anticoagulant use did not protect against development of severe COVID-19 [40]. Also, our preliminary analysis of the HOPE COVID-19 Registry observed a significantly lower survival and higher mortality risk in COVID-19 patients on OAC therapy at hospital admission compared to patients without prior OAC at admission [9]. More recently, a nationwide register-based cohort study in Sweden demonstrated that ongoing DOAC use at the time of SARS-CoV-2 infection was not associated with reduced risk of COVID-19 hospitalization or the composite of ICU admission or death due to COVID-19, indicating that the evidence for DOACs in this context is controversial [41].

Our results in the present study confirm our previous observation about the higher risk of mortality in COVID-19 patients with OAC therapy before hospital admission. Of note, our analysis is balanced by PSM, and there were no differences regarding admission to the ICU in patients on prior and no prior OAC. However, not only mortality was increased in patients with prior OAC therapy but also other clinical outcomes. Despite an appropriate PSM adjusting for comorbidities, postadmission serum creatinine as a marker of renal function (and injury) and troponins as markers of myocardial damage were higher in these patients, thereby showing increased rates of heart failure and renal failure during hospitalization. This reinforces the hypothesis that OAC-treated patients are particularly vulnerable and still have an inherent proinflammatory state.

### 4.1. Limitations

We should acknowledge some limitations in relation to this study. First, the constraints of an observational registry study of this design need to be considered. Second, the HOPE Registry only included patients from the first wave of the pandemic, and therefore, our results probably require further investigation during the subsequent waves. A bias inherent in the first wave neither can be excluded, given that hospitalization services throughout the world were overwhelmed. We also recognize that including several different indications for OAC may hinder and dissipate the specific effect that each indication has, since patients presented different risk profiles.

In addition, the indication for OAC as a whole may have some influence on the risk of outcomes, but comparing patients with prior OAC and no OAC was actually our aim, so we cannot adjust for specific indications for OAC but only for demographics data and other comorbidities at baseline. The absence of INR determinations (and therefore the time in therapeutic range (TTR)) in VKA-treated patients is also a limitation since the efficacy and safety of VKA depend on the quality of anticoagulant control, as reflected by the average TTR of INRs 2.0-3.0, and therefore may be related to the risk of worse outcomes. In addition, the type of DOAC was unknown in some cases, and this prevented us for analyzing drug types as separate. Finally, although this cohort was collected in a prospective manner, the results reported in this study are based on a post hoc analysis and should be regarded as hypothesis-generating.

## 5. Conclusion

Hospitalized COVID-19 patients on prior OAC therapy had a higher risk of mortality and worse clinical outcomes compared to patients without prior OAC therapy, even after adjusting for comorbidities using PSM. There were no differences in clinical outcomes in patients previously taking VKAs versus DOACs.

## Figures and Tables

**Table 1:** Comparison of clinical characteristics of the study cohort before and after propensity score matching.

	Before propensity score matching			After propensity score matching		
	Patients without prior OAC	Patients with prior OAC	*P* value	Patients without prior OAC	Patients with prior OAC	*P* value
	*N* = 7023	*N* = 675		*N* = 638	*N* = 638	
Demographic						
Male sex, *n* (%)	4097 (58.3)	403 (59.7)	0.491	386 (60.5)	372 (58.3)	0.425
Age (years), median (IQR)	63 (50–75)	80 (72–86)	<0.001	80 (72–86)	80 (72–86)	1.000
Race (non-Caucasian), *n* (%)	1603 (22.8)	59 (8.7)	<0.001	49 (7.7)	59 (9.2)	0.315
Body mass index (kg/m^2^), median (IQR)	27.1 (24.2–30.7)	27.7 (25.0–31.2)	0.011	26.9 (24.5–30.5)	26.7 (25.0–31.3)	0.168
Baseline comorbidities, *n* (%)						
Hypertension	3176 (45.2)	542 (80.3)	<0.001	433 (68.0)	516 (80.9)	0.053
Diabetes mellitus	1257 (17.9)	198 (29.3)	<0.001	168 (27.0)	190 (30.3)	0.198
Heart failure	128 (1.8)	46 (6.8)	<0.001	35 (5.5)	39 (6.1)	0.632
Stroke/TIA	439 (6.3)	131 (19.4)	<0.001	92 (14.4)	122 (19.1)	0.437
Chronic kidney disease	369 (5.3)	115 (17.0)	<0.001	69 (11.0)	109 (17.0)	0.487
Vascular disease^*∗*^	543 (7.7)	102 (15.1)	<0.001	93 (14.6)	88 (13.8)	0.688
Hypercholesterolemia	2096 (29.8)	344 (51.0)	<0.001	288 (45.1)	326 (51.1)	0.085
Current smoking habit	407 (5.8)	35 (5.2)	0.243	21 (3.3)	31 (4.9)	0.071
COPD/SAHS	419 (6.0)	104 (15.4)	<0.001	81 (12.7)	84 (13.2)	0.802
History of malignant disease	822 (11.7)	139 (20.6)	<0.001	129 (20.2)	129 (20.2)	1.000
Liver disease	238 (3.4)	33 (4.9)	0.001	30 (4.7)	31 (4.9)	0.795
Dysthyroidism	334 (4.8)	40 (5.9)	0.177	37 (5.8)	40 (6.3)	0.724
Any dependency level	819 (11.7)	210 (31.1)	<0.001	177 (28.2)	194 (30.6)	0.365
Concomitant treatment at admission, *n* (%)						
Beta-blockers	865 (12.3)	328 (48.6)	<0.001	132 (20.7)	311 (48.7)	<0.001
ACEi/ARBs	2320 (33.0)	369 (54.7)	<0.001	311 (48.7)	350 (54.9)	0.086
Antiplatelet therapy	1229 (17.5)	74 (11.0)	<0.001	199 (31.2)	72 (11.3)	<0.001
Laboratory parameters at admission						
Creatinine (mg/dL), median (IQR)	0.90 (0.72–1.17)	1.19 (0.90–1.64)	<0.001	0.98 (0.78–1.42)	1.20 (0.88–1.66)	<0.001
Hemoglobin (g/dL), median (IQR)	14.0 (12.0–15.0)	13.0 (11.0–14.0)	<0.001	13.0 (12.0–15.0)	13.0 (11.0–14.0)	<0.001
Platelet count (×10^9^/L), median (IQR)	203.0 (155.0–265.8)	179.0 (136.0–240.0)	<0.001	195.0 (145.0–260.8)	181.0 (138.0–241.0)	0.019
Elevated D-dimer, *n* (%)	3921 (55.8)	358 (53.0)	0.036	425 (66.6)	342 (53.6)	<0.001
Elevated procalcitonin, *n* (%)	1048 (14.9)	126 (18.7)	0.001	103 (16.1)	123 (19.3)	0.299
Elevated C-reactive protein, *n* (%)	5841 (83.2)	608 (90.1)	<0.001	566 (88.7)	576 (90.3)	0.657
Elevated troponins, *n* (%)	527 (7.5)	107 (15.9)	<0.001	54 (8.5)	100 (15.7)	<0.001
Elevated transaminases, *n* (%)	2598 (37.0)	220 (32.6)	0.009	216 (33.9)	210 (32.9)	0.023
Elevated ferritin, *n* (%)	2306 (32.8)	207 (30.7)	0.424	198 (31.0)	198 (31.0)	1.000
Elevated lactate dehydrogenase, *n* (%)	4414 (62.9)	464 (68.7)	0.005	427 (66.9)	440 (69.0)	0.466

ACEi, angiotensin-converting enzyme inhibitors; ARBs, angiotensin II receptor blockers; IQR, interquartile range; TIA, transient ischemic attack; COPD/SAHS, chronic obstructive pulmonary disease/sleep apnea-hypopnea syndrome. ^*∗*^Coronary artery disease and/or peripheral artery disease.

**Table 2:** Clinical outcomes during hospitalization after propensity score matching.

	Patients without prior OAC (*N* = 638)		Patients with prior OAC (*N* = 638)		OR (95% CI)	*P* value
	*N* (%)	Incidence per 100 patients-days (95% CI)	*N* (%)	Incidence per 100 patients-days (95% CI)		
Intensive care unit admission	40 (6.3)	0.52 (0.37–0.71)	44 (6.9)	0.58 (0.42–0.77)	1.11 (0.71–1.73)	0.652
Renal failure	151 (23.7)	1.97 (1.67–2.31)	212 (33.2)	2.77 (2.41–3.17)	1.61 (1.26–2.06)	0.001
Respiratory insufficiency	413 (64.7)	5.39 (4.89–5.94)	429 (67.2)	5.60 (5.09–6.16)	1.09 (0.86–1.38)	0.280
Upper respiratory tract infection	90 (14.1)	1.18 (0.95–1.44)	89 (13.9)	1.16 (0.93–1.43)	0.99 (0.72–1.35)	0.987
Heart failure	65 (10.2)	0.85 (0.66–1.08)	115 (18.0)	1.50 (1.24–1.80)	1.93 (1.39–2.68)	<0.001
Sepsis	77 (12.1)	1.01 (0.79–1.26)	96 (15.0)	1.25 (1.02–1.53)	1.29 (0.93–1.78)	0.299
Systemic inflammatory response syndrome	129 (20.2)	1.69 (1.41–2.00)	181 (28.4)	2.36 (2.03–2.73)	1.55 (1.20–2.02)	0.003
All-cause mortality	197 (30.9)	2.57 (2.23–2.96)	243 (38.1)	3.17 (2.79–3.60)	1.38 (1.09–1.74)	0.007
Any thrombotic/thromboembolic event	19 (3.0)	0.25 (0.15–0.39)	21 (3.3)	0.27 (0.17–0.42)	1.11 (0.59–2.08)	0.748
Any clinically relevant bleeding	26 (4.1)	0.34 (0.22–0.50)	73 (11.4)	0.96 (0.75–1.20)	3.04 (1.92–4.83)	<0.001

**Table 3:** Comparison of clinical characteristics of patients on VKA or DOAC prior admission after propensity score matching.

	Patients on prior VKA	Patients on prior DOAC	*P* value
	*N* = 232	*N* = 232	
Demographic			
Male sex, *n* (%)	139 (59.9)	127 (54.7)	0.260
Age (years), median (IQR)	80 (72–87)	81 (73–86)	0.575
Body mass index (kg/m^2^), median (IQR)	28.0 (25.1–31.6)	27.3 (24.3–31.0)	0.445
Baseline comorbidities, *n* (%)			
Hypertension	189 (81.5)	178 (76.7)	0.209
Diabetes mellitus	61 (26.3)	74 (31.9)	0.184
Heart failure	15 (6.5)	14 (6.0)	0.848
Stroke/TIA	42 (18.1)	47 (20.3)	0.555
Chronic kidney disease	31 (13.4)	31 (13.4)	1.000
Vascular disease^*∗*^	33 (14.2)	34 (14.7)	0.895
Hypercholesterolemia	112 (48.3)	113 (48.7)	0.926
Current smoking habit	13 (5.6)	9 (3.9)	0.143
COPD/SAHS	30 (12.9)	29 (12.5)	0.889
History of malignant disease	38 (16.4)	38 (16.4)	1.000
Dysthyroidism	18 (7.8)	17 (7.3)	0.860
Any dependency level	68 (29.3)	72 (31.0)	0.686
Concomitant treatment at admission, *n* (%)			
Beta-blockers	103 (44.4)	124 (53.4)	0.042
ACEi/ARBs	130 (56.0)	123 (53.0)	0.703
Antiplatelet therapy	24 (10.3)	27 (11.6)	0.656
Laboratory parameters at admission			
Creatinine (mg/dL), median (IQR)	1.19 (0.87–1.56)	1.13 (0.87–1.56)	0.628
Hemoglobin (g/dL), median (IQR)	13.0 (12.0–14.0)	13.0 (11.0–14.0)	0.853
Platelet count (×10^9^/L), median (IQR)	178.0 (138.0–244.8)	176.0 (134.0–233.0)	0.432
Elevated D-dimer, *n* (%)	121 (52.2)	118 (50.9)	0.954
Elevated procalcitonin, *n* (%)	45 (19.4)	40 (17.2)	0.795
Elevated C-reactive protein, *n* (%)	209 (90.1)	210 (90.5)	0.984
Elevated troponins, *n* (%)	35 (15.1)	31 (13.4)	0.711
Elevated transaminases, *n* (%)	86 (37.1)	66 (28.4)	0.138
Elevated ferritin, *n* (%)	75 (32.3)	61 (26.3)	0.344
Elevated lactate dehydrogenase, *n* (%)	167 (72.0)	150 (64.7)	0.182

ACEi, angiotensin-converting enzyme inhibitors; ARBs, angiotensin II receptor blockers; IQR, interquartile range; TIA, transient ischemic attack; COPD/SAHS, chronic obstructive pulmonary disease/sleep apnea-hypopnea syndrome. ^*∗*^Coronary artery disease and/or peripheral artery disease.

## Data Availability

The data used to support this study cannot be shared for ethical/privacy reasons.

## Supplementary Materials

Supplementary Materials On behalf of HOPE COVID-19 investigators, full list of investigators is shown in the Supplementary Material (HOPE participating hospitals, principal investigators, HOPE participating hospitals, coprincipal investigators, scientific committee, and list of collaborators). Supplementary Table 1. Clinical outcomes during hospitalization after propensity score matching in patients on prior oral anticoagulation therapy.
