# Supplementary material for: Chronic Oral Anticoagulation Therapy and Prognosis of Patients Admitted to Hospital for COVID-19: Insights from the HOPE COVID-19 Registry
Source: Int J Clin Pract. 2022 May 26;2022:7325060. doi: 10.1155/2022/7325060 (PMC9158796; doi:10.1155/2022/7325060)
Supplement: Supplementary Materials — On behalf of HOPE COVID-19 investigators, full list of investigators is shown in the Supplementary Material (HOPE participating hospitals, principal investigators, HOPE participating hospitals, coprincipal investigators, scientific committee, and list of collaborators). Supplementary Table 1. Clinical outcomes during hospitalization after propensity score matching in patients on prior oral anticoagulation therapy. [file 7325060.f1.docx]

**Supplementary Material**

**HOPE Participating Hospitals. Principal Investigators.**

| **NAME** | **SURNAME** | **HOSPITAL** | **DEPARTMENT** | **CITY** | **COUNTRY** |
| --- | --- | --- | --- | --- | --- |
| Vicente | Estrada | Hospital Clínico San Carlos | Internal Medicine | Madrid | Spain |
| Charbel | Maroun Eid | Hospital Universitario La Paz, Instituto de Investigación Hospital Universitario La Paz (IDIPAZ). | Emergency Department | Madrid | Spain |
| Rodolfo | Romero | Hospital Universitario Getafe | Emergency Department | Getafe | Spain |
| Inmaculada | Fernández Rozas | Hospital Universitario Severo Ochoa | Cardiology | Leganés | Spain |
| María C | Viana-Llamas | Hospital Universitario Guadalajara | Cardiology | Guadalajara | Spain |
| Victor Manuel | Becerra-Muñoz | Hospital Clínico Universitario Virgen de la Victoria | Cardiology | Malaga | Spain |
| Aitor | Uribarri | Hospital Clínico Universitario de Valladolid | Cardiology | Valladolid | Spain |
| Jia | Huang | The Second People's Hospital of Shenzhen | Critical care medicine | Shenzhen | China |
| Marcos | García Aguado | Hospital Puerta de Hierro de Majadahonda | Cardiology | Majadahonda | Spain |
| Emilio | Alfonso | Institute of Cardiology and Cardiovascular Surgery | Hemodynamics and Interventional Cardiology | La Habana | Cuba |
| Martino | Pepe | Azienda ospedaliero-universitaria consorziale policlinico di Bari | Division of Cardiology, Department of Emergency and Organ Transplantation, University of Bari, Bari, | Bari | Italy |
| Alex Fernando | Castro Mejía | Hospital General del norte de Guayaquil IESS Los Ceibos | Cardiology | Guayaquil | Ecuador |
| Fabrizio | Ugo | Sant'Andrea Hospital | Cardiology | Vercelli | Italy |
| Sergio | Raposeiras Roubin | University Hospital Álvaro Cunqueiro | Cardiology | Vigo | Spain |
| Jaime | Signes-Costa | Hospital Clínico | Neumology | Valencia | Spain |
| Carolina | Espejo Paeres | Hospital Universitario Príncipe de Asturias | Cardiology | Madrid | Spain |
| Gisela | Feltes Guzmán | NUESTRA SEÃ‘ORA DE AMERICA | Cardiology | Madrid | Spain |
| Enrico | Cerrato | San Luigi Gonzaga University Hospital, Orbassano and Rivoli Infermi Hospital, Rivoli (Turin), Italy | Interventional Cardiology | Turin | Italy |
| Francisco | Marín | Hospital Clínico Universitario Virgen de la Arrixaca | Department of Cardiology | Murcia | Spain |
| Javier | Lopez-Pais | Complejo Hospitalario Universitario de Santiago de Compostela | Cardiology | Santiago de Compostela | Spain |
| Luis | Buzón | Hospital Universitario de Burgos | Internal Medicine | Burgos | Spain |
| Daniela | Trabattoni | Centro Cardiologico Monzino, IRCCS | Invasive Cardiology | Milan | Italy |
| Thamar | Capel Astrua | Hospital Virgen del Mar | Internal Medicine | Madrid | Spain |
| Adelina | Gonzalez | Hospital Universitario Infanta Sofía | Anestesiology | San Sebastián de los Reyes | Spain |
| Jorge Luis | Jativa Mendez | Hospital de Especialidades de las Fuerzas Armadas N1 | Cardiology | Quito | Ecuador |
| Ibrahim | El-Battrawy | University Medical Center Mannheim | First Department of Medicine | Mannheim | Germany |
| Federico | Guerra | Ospedali Riuniti "Umberto I - Lancisi - Salesi" | Cardiology and Arrhythmology Clinic | Ancona | Italy |
| Alfredo | Bardaji | Joan XXIII | Cardiology | Tarragona | Spain |
| Miguel | Corbi-Pascual | Hospital General de Albacete | Cardiology | Albacete | Spain |
| Oscar | Fabregat-Andres | Hospital IMED Valencia | Cardiologia | Valencia | Spain |
| Maurizio | Bertaina | Martini Hospital. | Emergency Medicine Department | Turin | Italy |
| Christoph | Liebetrau | Kerckhoff Heart and Thorax Center | Cardiology | Bad Nauheim | Germany |
| Francesco | Santoro | ASL BAT | Cardiology | Andria | Italy |
| Bernardo Cortese | Cortese | San Carlo Clinic | Cardiology | Milano | Italy |
| Enrico | Pinotti | Policlinico San Pietro | Department of Surgery | Ponte San Pietro | Italy |
| Fabrizio D'Ascenzo | D'Ascenzo | San Giovanni Battista | Division of Cardiology | Torino | Italy |
| Andrea | Rognoni | A.O.U. Maggiore dellaq Carita , novara | Cardiology | Novara | Italy |
| Rodrigo | Bagur | London Health Sciences Centre | Medicine, Cardiology Division | London | Canada |
| Danilo | Buonsenso | Fondazione Policlinico Universitario A Gemelli IRCCS | Woman and Child Health and Public Health | Rome | Italy |
| Jose | moreu | complejo hospitalario de toledo | interventional cardiology | Toledo | Spain |
| Javier | Lopez-Pais | Hospital Clínico de Santiago de Compostela | Cardiology | Santiago de Compostela | Spain |
| Massimo | Mancone | Policlinico Umberto I | Cardiology | Rome | Italy |
| Mario | Iannaccone | San Giovanni Bosco, ASL CittÃ  di Torino, Turin, Italy | Cardiology | Turin | Italy |
| Alvaro | López Masjuan | Hospital Universitario Juan Ramón Jimenez | Cardiology | Huelva | Spain |
| Juan Fortunato | García Prieto | Hospital de Manises | Emergency Medicine | Valencia | Spain |
| Daniela | Coca | Hospital de Quilpue | Internal medicine | Quilpue | Chile |
| Jorge Luis | Velez | Hospital Pablo Arturo Suárez | Intensive Care Medicine | Quito | Ecuador |
| María Verónica | López Miao | Hospital de Especialidades Carlos Andrade Marín | Cardiology | Quito | Ecuador |
| Paola | Sanchez | Clinica CardioVID |  | Medellín | Colombia |

**HOPE Participating Hospitals. Co-Principal Investigators.**

| **NAME** | **SURNAME** | **HOSPITAL** | **DEPARTMENT** | **CITY** | **COUNTRY** |
| --- | --- | --- | --- | --- | --- |
| Angel | Molino | Hospital Clínico San Carlos | Internal Medicine | Madrid | Spain |
| Lubna | Dani Ben Abdellah | Hospital Universitario La Paz | Emergency Department | Madrid | Spain |
| Edoardo | Manzone | Hospital Universitario Getafe | Emergency Department | Getafe | Spain |
| María | Molina Villar | Hospital Universitario Severo Ochoa | Cardiology | Leganés | Spain |
| Ramón | Arroyo-Espliguero | Hospital Universitario Guadalajara | Cardiology | Guadalajara | Spain |
| Juan José | Gómez-Doblas | Hospital Clínico Universitario Virgen de la Victoria | Cardiology | Malaga | Spain |
| Álvaro | Aparisi | Hospital Clínico de Valladolid | Cardiology | Valladolid | Spain |
| Lei | Liu | The Third People's Hospital of Shenzhen | General Surgery | Shenzhen | China |
| Juan Antonio | Vargas Nuñez | Hospital Puerta de Hierro de Majadahonda | Medicina Interna | Majadahonda | Spain |
| Gaetano | Brindicci | Azienda ospedaliero-universitaria consorziale policlinico di Bari | Clinic of Infectious Diseases, Hospital-University Polyclinic, University of Bari, Bari, Italy | Bari | Italy |
| Maria Elizabeth | Ortega Armas | Hospital General del norte de Guayaquil IESS Los Ceibos | Cardiology | Guayaquil | Ecuador |
| Francesco | Rametta | Sant'Andrea Hospital | Cardiology | Vercelli | Italia |
| Karim | Jamhour Chelh | University Hospital Álvaro Cunqueiro | Cardiology | Vigo | Spain |
| Carmen | Castellanos-Lluch | Hospital Clínico | Medicina Interna | Valencia | Spain |
| Ana María | Carrero Fernández | Hospital Universitario PrÃ­ncipe de Asturias | Servicio de Urgencias | Madrid | Spain |
| Asunción | Guerri | Nuestra Señora de América | Internal Medicine | Madrid | Spain |
| Matteo | Bianco | San Luigi Gonzaga University Hospital, Orbassano | Cardiology | Turin | Italy |
| Elisa | García-Vazquez | Hospital Clínico Universitario Virgen de la Arrixaca | Department of Internal Medicine-Infectious Diseases | Murcia | Spain |
| José María | García-Acuña | Complejo Hospitalario Universitario de Santiago de Compostela | Cardiology | Santiago de Compostela | Spain |
| Jose Antonio | Fernandez-Ratero | Hospital Universitario de Burgos | Unidad de Cuidados Intensivos | Burgos | Spain |
| Emilio | Assanelli | Centro Cardiologico Monzino, IRCCS | Emergency care | Milan | Italy |
| Chinh | Pham Trung | Hospital Virgen del Mar | Cardiology | Madrid | Spain |
| Clara | Cimarra | Hospital universitario Infanta Sofia | Anestesiologia y reanimacion | Madrid | Spain |
| Ibrahim | Akin | University Medical Center Mannheim | First Department of Medicine | Mannheim | Germany |
| Antonio | Dello Russo | Ospedali Riuniti "Umberto I - Lancisi - Salesi" | Cardiology and Arrhythmology Clinic | Ancona | Italy |
| Teresa | Auguet | Joan XXIII | Cardiology | Tarragona | Spain |
| Juan Gabriel | Cordoba-Soriano | Hospital General de Albacete | Cardiology | Albacete | SPain |
| Fabio | De Iaco | Martini Hospital | Emergency Medicine Department | Turin | Italy |
| Sergio | Carbonara | Bisceglie | Infectious Disease | Bisceglie | Italy |
| Gaetano | Di Palma | San Carlo Clinic | Cardiac | Milano | Italy |
| Davide | Pata | Policlinico Universitario Agostino Gemelli IRCSS | Department of Woman and Child Health and Public Health | Rome | Italy |
| Diego | Lopez-Otero | Hospital Clínico de Santiago de Compostela | Cardiology | Santiago de Compostela | Spain |
| Fabio | Infusino | Policlinico Umberto I | Cardiology | Rome | Italy |
| Giacomo | Boccuzzi | San Giovanni Bosco, ASL Citta di Torino | Cardiology | Turin | Italy |
| Adrían | Rodriguez Albarran | Hospital Universitario Juan Ramón Jimenez | Cardiology | Huelva | Spain |
| Diana Isabel | Salazar Chamba | Hospital de Especialidades Carlos Andrade Marín | Cardiology | Quito | Ecuador |

**Scientific Committee**

| NAME | FILIATION | COUNTRY |
| --- | --- | --- |
| Iván J. Núñez-Gil | Hospital Clínico San Carlos, Madrid | Spain |
| Carlos Macaya | Hospital Clínico San Carlos, Madrid | Spain |
| Asunción Guerri | Hospital Nuestra Señora de América, Madrid | Spain |
| Charles Lefranc | Hospital Nuestra Señora de América, Madrid | Spain |
| David Orgaz | Primary Care, Madrid | Spain |
| Enma Gil Higes | Primary Care, Madrid | Spain |
| Juan Conesa | Hospital Clínico San Carlos, Madrid | Spain |
| Antonio Fernández Ortiz | Hospital Clínico San Carlos, Madrid | Spain |
| Harish Ramakhrisna | Mayo Clinic, Rochester | United States |
| Julio Jiménez | SUMMA 112, Madrid | Spain |
| Vanessa García de Biedma | Hospital Universitario de Fuenlabrada | Spain |
| Ruth Sendino | Primary Care, Vitoria | Spain |
| Gisela Feltes | Hospital Nuestra Señora de América, Madrid | Spain |
| Sergio Raposeiras | Hospital Álvaro Cunqueiro, Vigo | Spain |
| Giusseppe Biondi Zoccai | Sapienza University of Rome, Latina, Italy | Italy |
| Ángel Molino | Hospital Clínico San Carlos, Madrid | Spain |
| Cristina Fernández | Hospital Clínico San Carlos, Madrid | Spain |
| Vicente Estrada | Hospital Clínico San Carlos, Madrid | Spain |
| Fabrizio D´Ascenzo | Città della Salute e della Scienza, Turin | Italy |
| Enrico Cerrato | San Luigi Gonzaga University Hospital, Orbassano and Rivoli Infermi Hospital, Rivoli (Turin), Italy | Italy |
| F. Javier Martín-Sánchez | Hospital Clínico San Carlos, Madrid | Spain |

**List of collaborators**

| Centre | City | Country | Name |
| --- | --- | --- | --- |
| San Carlo Clinic | Milano | Italy |  |
| Hospital Clínico Universitario de Valladolid | Valladolid | Spain |  |
| London Health Sciences Centre | London | Canada |  |
| Hospital IMED Valencia | Valencia | Spain |  |
| Ospedali Riuniti "Umberto I - Lancisi - Salesi" | Ancona | Italy | Giulia Stronati |
| Institute of Cardiology and Cardiovascular Surgery | La Habana | Cuba | Emilio Alfonso  Giselle Lopez |
| Hospital Clínico Universitario Virgen de la Victoria | Málaga | Spain | Eva Cabrera-Cesar  Belén Murcia-Casas  Álvaro Martínez-Mesa  Esther Sanchez-Alvarez  Victoria Doncel-Abad  Pilar Nuevo-Ortega  Carmen Perez-Lopez  Guillermo Ojeda-Burgos  Javier Sanchez-Lora  Jesus Santos-Gonzalez |
| Centro Cardiologico Monzino, IRCCS | Milano | Italy | Francesca Susini  Giulia Santagostino  Sebastiano Gili  Giovanni Teruzzi  Massimo Mapelli  Ricardo Maragna  Irene Mattavelli  Elisabetta Salvioni |
| Complejo Hospitalario de Toledo | Toledo | Spain | Tomas Canton  Esther Lazaro  Luis Manuel Hernando  Luis Fernando Pajin |
| San Giovanni Battista | Torino | Italy | Ovidio De Filippo  Guglielmo Gallone  Francesco Bruno  Gaetano Maria De Ferrari  Luca Franchin  Filippo Angelini  Pierluigi Omedé  Federico Conrotto  Mauro Pennone  Antonio Montefusco |
| Policlinico A. Gemelli IRCSS | Rome | Italy | Davide Pata  Danilo Buosenso  Piero Valentini  Antonio Gatto  Antonio Chiaretti |
| Hospital Nuestra Señora de América | Madrid | Spain | Carmen Díaz Sánchez.  José Luis Moreno Hurtrez  Charles Lefranc.  María Luisa Sheriff Prosper. |
| Policlinico Umberto I | Rome | Italy | Ilaria Birtolo  Paolo Severino  Francesco Pugliese  Francesco Fedele  Gabriella d'Ettorre  Gioacchino Galardo  Claudio Maria Mastroianni  Niccola Salvi  Sara Cimino  Carlo Lavalle |
| University Medical Center Mannheim | Mannheim | Germany | Ibrahim El-Battrawy  Mohammad Abumayyaleh  Ibrahim Akin |
| The Second People's Hospital of Shenzhen | Shenzhen | China | Fulan Cen  Song Wang  Manfei Zeng  Liping Qiu  Fangfan Ye  Yajing Huang |
| Complejo Hospitalario Universitario de Santiago de Compostela | Santiago de Compostela | Spain | Diego Lopez-Otero  Taboada Muñiz |
| Hospital Universitario Guadalajara | Guadalajara | Spain |  |
| Hospital Clínico de Valencia | Valencia | Spain | María Jose Forner  Vicente Bodi  Elvira Bondia  Paloma Albiol  Nerea Perez-Solé  Alba Mulet  Ana de Gracia  Lucía Fernández—Presa  Clara Bonanad  Joaquin Canoves |
| Hospital Clínico San Carlos | Madrid | Spain | Oscar Vedia  Carlos Vergara  Víctor Hugo Moreno Munguía  Francisco Javier Martin  José Tomás Ramos  Miriam de La Puente Yague  Juana María Brenes Sánchez  Inés Gil Prados  Isabel Fernández  María Arantzazu Álvarez de Arcaya  María Ángeles Cuadrado  Carlos Elvira  Andrea Valcárcel Alonso,  María del Rosario Iguaran Bermúdez,  Carmen Blasco-Fanlo  Cristina Villamor Jimenez,  Cristina Botella Moreno,  ErnestoMora Fernández,  Jesús Jacob  Carlos Ignacio Garcia-Asenjo,  Carmen Cuervo Molinero,  Iñigo Sagastagoitia Fornie,  Federico Cuesta Triana,  Juncal Perez- Somarriba,  Angel Nieto Sánchez,  Lara Guardado Fuentes |
| Hospital Severo Ochoa | Leganés | Spain | María Molina |
| Hospital Joan XXIII | Tarragona | Spain |  |
| San Luigi Gonzaga University Hospital, Orbassano and Rivoli Infermi Hospital, Rivoli (Turin), Italy | Turin | Italy | Amanda Spirito  Flavio Pietrangiolillo  Salvatore Campagnuolo |
| Hospital General del norte de Guayaquil IESS Los Ceibos | Guayaquil | Ecuador | Erick Alexander Jara León  Galo Guillermo Farfán Cano  Fernando Aníbal Marmol Mosquera  Luis ALfonso Moreno Rondón  Diego Raúl Villavicencio García  Eric Antonio Alava Montesdeoca  José Luis Rodríguez Campos |
| Hospital Clínico Universitario Virgen de la Arrixaca | Murcia | Spain | José Miguel Rivera-Caravaca  Pablo Gil-Pérez  Cecilia López-García  Domingo Pascual-Figal  Antonio Tello-Montoliu  Encarnación Moral Escudero  Alicia Hernández Torres  Aychel Elena Roura Piloto |
| Hospital Virgen del Mar | Madrid | Spain | Oriol Casals Rafecas  Victoria Márquez Fernández  María Jesús González Juárez  Ada Viviana Romero Echeverry  Paola Tatiana García Giraldo  Carlos de la Fuente Gutiérrez  Manuel Montes Lluch  María Eugenia García Ramírez  Joaquín Solís Jiménez  Reina García Closas |
| Azienda ospedaliero-universitaria consorziale policlinico di Bari | Bari | Italy | Palma Luisa Nestola  Maria Grazia Carmela La Marca  Gioacchino Angarano  Annalisa Saracino  Onofrio Resta  Giovanna Elisiana Carpagnano  Enrico Buonamico  Salvatore Grasso  Lidia Dalfino  Rachele Iannuzziello |
| Hospital Universitario de Getafe | Madrid | Spain | Virginia Álvarez  Silvia Bleda  Silvia Odeh  Gema Núñez  María Isabel Ortega  Joaquín Jesús Alonso  Alfonso Fraile |
| University Hospital Álvaro Cunqueiro | Vigo | Spain | Emad Abu Assi  Andrés Iñiguez Romo  Dolores Vila  María Teresa Pérez Rodriguez  Alexandre Perez |
| Hospital Universitario La Paz | Madrid | Spain | María Angélica Rivera Núñez  Clara Cabre-Verdiell Surribas  Victoria Lo Iacono García  Rosario Torres Santos Olmo  Berta Anton Huguet  Isabel Arenas Berenguer  Berta Alonso Gonzalez  Begoña Reche Martínez  Regina Cabrera Gamero  Belén Oliva  Mikel Rico Brías  Lorena López Corcuera |
| Martini Hospital,via Tofane | Turin | Italy | Francesco Fioravanti  Luca Franchin |
| San Giovanni Bosco, ASL Citta di Torino | Turin | Italy | Umberto Annone |
| A.O.U. Maggiore dellaq Carita | Novara | Italy |  |
| ASL BAT | Andria | Italy |  |
| Hospital de Especialidades de las Fuerzas Armadas N1 | Quito | Ecuador | María Belén Diaz  Diana Salgado  Santiago Andrés Gualivisi |
| Hospital General de Albacete | Albacete | Spain | Juan Gabriel Córdoba Soriano  Raquel Ramos Martinez  Laura Expósito Calamardo |
| Hospital Puerta de Hierro de Majadahonda | Majadahonda | Spain | Marcos García Aguado  Juan Antonio Vargas Núñez  Patricia Serrano de la Fuente  Monica Sanchez Santiuste  Carmen Vizoso Galvez  Cristina Moreno López  Mercedes Valentín Pastrana Aguilar  Isabel Redondo Cánovas del Castillo  Lucia Romero Imaz  Claudia García Rodríguez Maimán  Jose María Camino Salvador  Miguel Del pino Jimenez |
| Hospital Universitario de Burgos | Burgos | Spain | Luis Buzón  Jose Antonio Fernández Ratero  Bibiana Carreira  Jose Ángel Pérez Ribera  Miguel Montero  Juan Manuel de Vicente  Juan Pablo Garcia Muñoz  María Fernandez Reguereas  María López Veloso  Carolina Navarro  Jorge Boado  Sergio Ossa |
| Hospital Universitario Infanta Sofía | San Sebastián de los Reyes | Spain | Adelina Gonzalez  Clara Cimarra  María Barrionuevo  Jesus Varas  Rita Esther Medina  Paloma Mateo  Carmen Rodriguez |
| Hospital Universitario Príncipe de Asturias | Madrid | Spain | Carolina Espejo Paeres  Ana María Carrero Fernández  Honan Roiz Andino  Carolina Aguilar Andrea  María Ángeles López López  Cesar Briega Iglesias  Fernando Calvo Garrido  María Isabel Carrasco  Angel Luis Martín Sonseca  Wael Humaid  Gema Esteban Gutierrez  María Tuian Leiva |
| Kerckhoff Heart and ManiesThorax Center | Bad Nauheim | Germany | Cristoph Liebetrau  J Wolter  Andreas Rolf |
| Policlinico San Pietro | Ponte San Pietro | Italy | Enrico Pinotti |
| Hospital Universitario Juan Ramón Jimenez | Huelva | Spain | Alvaro López Masjuan  Adrián Rodríguez Albarrán  Santiago Jesus Camacho Freire  Maria Isabel Cabello Lopez  Macarena Chávez Navarro  Manuel García de la Vega Sosa  Miguel Ángel Montilla Garrido  Carmen Lluch Requerey  Pedro Pájaro Merino  Samuel Ortiz cruces  Andrea Sigismondi |
| Hospital de Quilpue | Quilpue | Chile | Daniela Cocas |
| Hospital de Manises | Valencia | Spain | Juan Fortunato García Prieto  Inmaculada Atienza Garrido  Estefanía Tovar Jorge  Elisa Carmen Castillo Espinoza  Andrea Ivette Romero Carpio  Maria Martinez Fort  Rosa Ruiz  Patricia Sanchez |
| Hospital Pablo Arturo Suárez | Quito | Ecuador | Jorge Luis Velez  Edgar Lopez  Estefanía Irigoyen |
| Hospital de Especialidades Carlos Andrade Marín | Quito | Ecuador | María Verónica Lopez Miao  Diana Isabel Salazar Chamba  Freddy Peralta Coronel |
| London Health Sciences Centre | London | Canada | Rodrigo Bagur  Luciano Sposato |
| Clinica CardioVID | Medellin | Colombia | Paola Sanchez |

**Supplementary Table 1.** Clinical outcomes during hospitalization after propensity score matching in patients on prior oral anticoagulation therapy.

|  | **Patients on prior DOAC**  **(N = 232)** | | | **Patients on prior VKA**  **(N = 232)** | | **OR (95% CI)** | **p-value** |
| --- | --- | --- | --- | --- | --- | --- | --- |
|  | **N (%)** | **Incidence per 100 patients-days (95% CI)** | **N (%)** | | **Incidence per 100 patients-days (95% CI)** |  |  |
| Intensive care unit admission | 14 (6.0) | 0.50 (0.27-0.84) | 14 (6.0) | | 0.50 (0.27-0.84) | 1.00 (0.47-2.15) | 1.000 |
| Renal failure | 71 (30.6) | 2.55 (1.99-3.22) | 74 (31.9) | | 2.66 (2.09-3.34) | 1.12 (0.75-1.67) | 0.107 |
| Respiratory insufficiency | 154 (66.4) | 5.53 (4.69-4.48) | 155 (66.8) | | 5.57 (4.73-6.51) | 1.03 (0.70-1.53) | 0.932 |
| Upper respiratory tract infection | 32 (13.8) | 1.15 (0.79-1.62) | 29 (12.5) | | 1.04 (0.70-1.15) | 0.93 (0.54-1.60) | 0.287 |
| Heart failure | 40 (17.2) | 1.44 (1.03-1.96) | 38 (16.4) | | 1.36 (0.96-1.87) | 0.98 (0.60-1.59) | 0.223 |
| Sepsis | 35 (15.1) | 1.26 (0.88-1.75) | 41 (17.7) | | 1.47 (1.06-2.00) | 1.24 (0.76-2.04) | 0.347 |
| Systemic inflammatory response syndrome | 61 (26.3) | 2.19 (1.68-2.81) | 66 (28.4) | | 2.37 (1.83-3.02) | 1.14 (0.75-1.72) | 0.647 |
|  |  |  |  | |  |  |  |
| **All-cause mortality** | 92 (39.7) | 3.31 (2.66-4.05) | 88 (37.9) | | 3.16 (2.53-3.89) | 0.93 (0.64-1.35) | 0.703 |
| **Any thrombotic/thromboembolic event** | 3 (1.3) | 0.11 (0.02-0.31) | 8 (3.4) | | 0.29 (0.12-0.57) | 2.73 (0.71-10.41) | 0.127 |
| **Any clinically relevant bleeding** | 23 (9.9) | 0.83 (0.52-1.24) | 28 (12.1) | | 1.01 (0.67-1.45) | 1.25 (0.70-2.24) | 0.458 |
